# Beneath the Surface: Rethinking Concordance in Renal Imaging and Function for ADPKD

**DOI:** 10.1016/j.ekir.2025.07.048

**Published:** 2025-08-09

**Authors:** Zhen Li, Gang Wang

**Affiliations:** 1 Department of Medical Imaging, Nanjing Gaochun Hospital of Traditional Chinese Medicine, Nanjing, China

To the Editor:

We have read with great interest the recent study by Lee *et al.*,^1^ which investigated a unique clinical phenotype in autosomal dominant polycystic kidney disease (ADPKD)—patients with mild imaging findings, but a significantly reduced estimated glomerular filtration rate. The authors found that 12% of the 473 patients exhibited this imaging-function discordance and proposed potential etiological subtypes. This study provides valuable insights into the clinical heterogeneity of ADPKD; however, we would like to raise several points for further discussion.

First, the study exclusively employed the Chronic Kidney Disease-Epidemiology Collaboration 2012 equation for estimated glomerular filtration rate estimation, without considering the recently introduced European Kidney Function Consortium equation, which has demonstrated improved accuracy in certain populations.^2^ In addition, the study did not use cystatin C–based estimated glomerular filtration rate calculations, despite the established value of cystatin C in assessing kidney function in patients with abnormal muscle mass.^3^ These methodological limitations may affect the accurate characterization of imaging-function discordance in ADPKD.

Second, although the authors discuss the concept of cardiovascular-renal-metabolic syndrome, the assessment of metabolic syndrome did not follow established international criteria such as ATP III or IDF. Key indicators, including insulin resistance and waist circumference, were not collected, nor was the actual prevalence of metabolic syndrome reported.^4^ These limitations restrict the scientific evaluation and mechanistic understanding of metabolic factors in the pathogenesis of imaging-function discordance in ADPKD. Future studies should adopt standardized metabolic syndrome definitions and comprehensive assessments to facilitate more accurate risk stratification and personalized management.

In addition, the findings of this study have not been externally validated in independent cohorts across diverse ethnic and geographic populations. The prevalence and underlying causes of the “discordant phenotype” observed may be influenced by population-specific genetic and environmental factors.^5^ Although the authors acknowledge the potential for referral bias because of the single-center design, the absence of external validation remains a key limitation for the broader applicability of their conclusions.

In conclusion, despite needing further clarification on certain issues, Lee *et al.*’s^1^ research enhances understanding of the clinical importance of imaging and renal function discrepancies in patients with ADPKD, aiding in the development of precise individualized management strategies.

## Disclosure

All the authors declared no competing interests.

## Author Contributions

ZL contributed to the investigation and writing of original draft. GW contributed to supervision and writing (review and editing).
